# Effects of maternal *Echinococcus multilocularis* infection on colitis susceptibility and gut microbiota of offspring

**DOI:** 10.1186/s13071-025-06915-8

**Published:** 2025-07-26

**Authors:** Yihui Liu, Yang Xu, Yang Zou, Yingying Ding, Jiayun Zhang, Ying Zhang, Quanhai Pang, Shuai Wang

**Affiliations:** 1 College of Veterinary Medicine, Shanxi Agricultural University, Taigu, 030801 Shanxi China; 2 State Key Laboratory of Animal Disease Control and Prevention, College of Veterinary Medicine, Lanzhou University, Lanzhou Veterinary Research Institute, Chinese Academy of Agricultural Sciences, Lanzhou, 730000 Gansu China

**Keywords:** *Echinococcus multilocularis*, Colitis model, *Foxp3*, Gut microbiota, Maternal transmission

## Abstract

**Background:**

Maternal immune modulation and alterations in gut microbiota due to intestinal helminth infections may be passed on to offspring. However, it remains unclear whether these effects can be transferred between maternal mice and their offspring during tissue-dwelling helminth infections.

**Methods:**

In this study, we investigated the effect of maternal infection with *Echinococcus multilocularis* (*Emu*) on offspring susceptibility to colitis and gut microbiota composition using a dextran sulfate sodium (DSS)-induced colitis model. We performed 16S ribosomal RNA (rRNA) sequencing to analyze the gut microbiota composition and microbial abundance in *Emu*-infected and control maternal mice, as well as their offspring.

**Results:**

We found that the maternal mice infected with *Emu* exhibited significant resistance to colitis, characterized by increased expression of *Foxp3* in colonic tissue. Conversely, this resistance phenotype was not observed in the offspring of *Emu*-infected maternal mice, as they showed no reduction in colitis severity and demonstrated decreased *Foxp3* expression. Furthermore, the gut microbiota of *Emu*-infected maternal mice underwent significant changes, with an increase in genera such as *Rikenella*, *Rikenellaceae RC9 gut group*, *Turicibacter*, *Odoribacter*, and *Parabacteroides*, while *Lactobacillus*, *Staphylococcus*, and *Bifidobacterium* decreased postinfection. By contrast, their offspring exhibited a markedly distinct gut microbiota shift, characterized by significant increases in *Candidatus Saccharimonas*, *Desulfovibrio*, *Helicobacter*, and *Odoribacter*, alongside significant reductions in *Muribaculum* and *Clostridium *sensu stricto* 1* when compared with the offspring of naive mice.

**Conclusions:**

These findings suggest that the effects of maternal transmission concerning immune regulation and microbiota alterations in response to helminth infections may depend on species-specific factors.

**Graphical Abstract:**

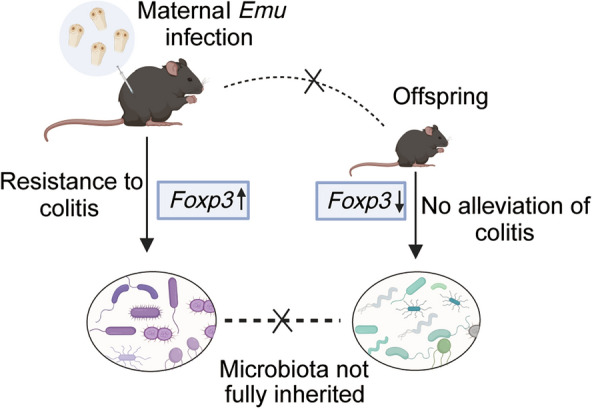

## Background

Helminth infections, affecting over 1.5 billion people globally, remain a major public health issue [1]. These parasitic infections not only are pervasive but also exhibit an inverse association with the prevalence of autoimmune conditions, such as allergies [2] and inflammatory bowel disease (IBD) [3, 4]. Despite species-specific differences, helminths modulate the host’s immune response to the parasites [5, 6]. Given that helminths can regulate host immune function—an effect that is partially reversible following anthelmintic treatment—there is growing interest in elucidating the mechanisms responsible for this immunomodulation, particularly the parasite-derived molecules that may mediate these effects.

Emerging evidence indicates that helminths possess the capacity to modulate host immune responses, potentially offering a novel approach to treating autoimmune diseases. Helminths regulate host immunity primarily through Th2 responses and regulatory T (Treg) cell expansion, which maintain immune homeostasis and suppress inflammation [7, 8]. *Foxp3*, a critical transcription factor within the forkhead box (Fox) family, is primarily expressed in CD4^+^ T cells, where it regulates the gene expression of Treg cells and is responsible for their suppressive function in immune regulation [9]. Recent studies increasingly indicate that helminth infection has the capacity to reshape the composition of the gut microbiome [10]. This crosstalk between gut microbiota and host immunity is essential for maintaining physiological stability and influencing disease outcomes. The gut microbiota composition, a crucial determinant of host health, is significantly influenced by specific microorganisms acquired early in life, particularly from the mother. Recent research indicates that alterations in gut microbiota induced by helminth infections can be transmitted from the mother to her offspring [11]. A previous study found that infection of the maternal mouse with *Heligmosomoides polygyrus* altered the maternal gut microbiota structure and inhibited obesity induced by high-fat diet. Remarkably, the offspring of these infected mice exhibited a similar gut microbiota profile to their mothers and demonstrated a similar resistance to diet-induced obesity [12]. IBD has been linked to significant alterations in gut microbiome structure and diversity, not only in affected individuals but also in their offspring. Recent studies have shown that transplanting gut microbiota from patients with IBD and their progeny into germ-free mice induces dysbiosis and abnormal immune responses in the recipients [13]. These findings highlight the critical role of early microbial exposure in shaping subsequent generations’ health outcomes and reveal the gut microbiota’s pivotal role in the intergenerational transmission of health benefits. However, the extent to which maternal helminth-induced immune modulation and microbiota alterations contribute to protecting offspring from colitis remains unclear, particularly for nonintestinal helminths such as *Echinococcus multilocularis* (*Emu*), which primarily inhabit extraintestinal tissues. This study explores the effects of maternal *Emu* infection on offspring gut microbiota and immune responses, focusing on their role in colitis resistance. Using a mouse model, we examined how maternal *Emu* infection modulates gut microbiota and *Foxp3* expansion and whether these effects are transmitted to offspring through the gut microbiota. By elucidating the mechanisms underlying maternal helminth-induced immune regulation, this study aims to provide insights into the intergenerational impact of helminth infections and explore potential therapeutic strategies for inflammatory diseases such as IBD in humans and animals.

## Methods

### Mice and infections

Specific-pathogen-free (SPF) female C57BL/6 mice, aged 7–9 weeks, were purchased from Changzhou Cavens Laboratory Animal Co., Ltd (Jiangsu, China). The animals were maintained under standard conditions with ad libitum water and chow access. In addition, mice were subjected to a controlled environment with regulated temperature and humidity, following a half-day light cycle. All animal use and experiments that received ethical approval, including the euthanasia of mice, were reviewed and approved by the Institutional Animal Care and Use Committee of Lanzhou Veterinary Research Institute (no. LVRIAEC-2020-009). All experiments were conducted in accordance with applicable ethical guidelines for the care and use of laboratory animals..

The *E. multilocularis* strain used in this study was isolated from Qinghai in China. The collection of protoscoleces (PSCs) was conducted as previously described [14, 15]. Parasite tissues were aseptically retrieved from the peritoneal cavity of gerbils (*Meriones unguiculatus*) that had received intraperitoneal inoculation of protoscoleces for 3 months. The parasitic cyst tissue was cut into thin slices and filtered through a double-layered copper mesh. Subsequently, the gathered PSCs underwent five washes with sterile phosphate-buffered saline (PBS) with penicillin (100 U/mL) and streptomycin (0.1 mg/mL). In each experimental trial, mice in the *Emu* group received an intraperitoneal injection of 2,000 PSCs suspended in 200 μL of PBS. In contrast, the naive mice received the same volume of PBS via intraperitoneal injection. Typically, cysts are located within the peritoneal cavity of the infected mice.

Female mice were pretreated with either *Emu* infection or PBS as a control before being paired with uninfected males for mating. Approximately 6 weeks post-*Emu* infection, female mice were cohoused with uninfected male mice at a ratio of 2:1. The control group was housed similarly. Female offspring mice were separated from their mothers at 21 days of age and subsequently maintained for 9 weeks.

### Dextran sodium sulfate-induced colitis and histology

The mouse model of dextran sulfate sodium (DSS)-colitis was established following previously described protocols [16]. DSS (MW 36,000–50,000) was dissolved in drinking water at a concentration of 3% (w/v), and the solution was refreshed every 2 days. Control mice were provided with sterilized drinking water without DSS. Successful induction of colitis was confirmed by consistent weight loss, the onset of diarrhea, and the appearance of fecal blood starting on the third day of DSS treatment. All mice were euthanized on day 8 following the start of DSS treatment for sample collection and analysis.

### Disease activity index (DAI) assessment

The DAI score was calculated daily to determine the severity and progression of intestinal inflammation in mice, based on body weight change, stool consistency, and the presence of fecal blood. Body weight reduction was scored as follows: 0, 0–1%; 1, 1–5%; 2, 5–10%; 3, 10–15%; and 4, 15–20%. Stool consistency was graded on a scale from 0 to 4: 0, normal; 1, softly formed; 2, soft unformed; 3, in-between; and 4, diarrhea. Fecal blood was scored as follows: 0, − ; 1, + ; 2, +  + ; 3, +  +  + ; 4, +  +  +  + . As described previously, the DAI score was determined by summing the scores for body weight loss, stool consistency, and fecal blood.

### Colon length and histopathology

After euthanizing the mice, colon length was measured. The distal colon tissue was preserved in 4% (v/v) formalin. Tissues were processed and stained with hematoxylin and eosin (H&E) to assess histological differences between experimental mice. The scoring process was conducted blinded and was based on predefined criteria as referenced [17]. The cellular infiltrate was scored as follows: 0 indicated occasional inflammatory cells in the lamina propria (LP); 1 indicated increased infiltrate in the LP, primarily at crypt bases; 2 represented confluent infiltrate extending into the mucosa; and 3 signified transmural extension. Tissue damage was evaluated using this scoring system: 0 indicated no mucosal damage, 1 indicated up to 50% crypt loss, 2 indicated 50-100% crypt loss with intact epithelium, and 3 indicated complete crypt and epithelial loss.

### Quantitative reverse transcription polymerase chain reaction (qRT-PCR)

Following euthanasia, colon tissues were collected for RNA extraction using the TRIzol reagent (Invitrogen, Carlsbad, CA) following the manufacturer’s protocol. The complementary DNA (cDNA) was generated using OligoDT primers and random primers through the GoScript Reverse Transcription System kit (Promega, USA). The expression level of *Foxp3* in colonic samples was quantified by qPCR using specific primers listed in Table 1. qPCR reactions were performed utilizing the SYBR Green-based method of the GoTaq^®^ qPCR Master Mix Kit (Promega, USA) under the recommended thermal cycling conditions (Tm = 60 °C) on an “Applied Biosystems 7500” Real-Time PCR system (ABI, Thermo Fisher Sci). Relative expressions were determined using the 2^−ΔΔ CT^ method with *b2m* used as the reference gene for normalization.

**Table 1:** qPCR primer sequence list

Primers	Forward primer sequence	Reverse primer sequence
Foxp3	ACCATTGGTTTACTCGCATGT	TCCACTCGCACAAAGCACTT
b2m	TTCTGGTGCTTGTCTCACTGA	CAGTATGTTCGGCTTCCCATTC

### 16S rRNA gene sequencing

The fecal DNA of mice was extracted using the DNA extraction kit (catalog number (cat. no.) DP328-02, Tiangen). Sequencing libraries were constructed according to Illumina’s protocol for bacterial 16S rRNA gene sequencing. Specifically, the V3–V4 hypervariable region of the 16S rRNA gene was amplified with primers 338F (5′-ACTCCTACGGGAGGCAGCAG-3′) and 806R (5′-GGACTACHVGGGTWTCTAAT-3′) using TransStart FastPfu DNA Polymerase (TransGen, Beijing, China). PCR amplicons were purified using the AxyPrep DNA Gel Extraction Kit (cat. no. AP-96-GX, AXYGEN) and quantified with the QuantiFluor™-ST system (Promega). Equal quantities of the purified amplicons were combined and subjected to sequencing on the Illumina MiSeq platform using the NEXTFLEX Rapid DNA-Seq Kit, in accordance with the manufacturer’s protocol.

Paired-end reads obtained from Illumina sequencing were first merged on the basis of overlapping sequences. Quality control and filtering were conducted to ensure high-quality sequences. The processed reads were then assigned to samples and clustered into operational taxonomic units (OTUs) using a 97% similarity threshold using Uparse (v11) and annotated to SILVA138 database using RDP Classifier (v2.13) (confidence threshold: 70%). The abundance for each OTU was estimated using QIIME (v1.9.1) with default settings.

### Statistical analysis

Statistical analyses were conducted using Prism 9 software (GraphPad Software, La Jolla, CA, USA). Comparisons of taxonomic abundance and alpha diversity metrics between groups were conducted using the two-sided Wilcoxon rank-sum test with a 95% confidence interval. Principal coordinate analysis (PCoA) of β-diversity was performed using permutational multivariate analysis of variance (PERANOVA) through the Adonis2 function in the vegan package (v2.6–4) in R. Group comparisons were analyzed using an unpaired Student’s *t*-test for between-group comparisons or two-way repeated analysis of variance (ANOVA) followed by Sidak’s posttest. For multiple-group comparisons, the Kruskal–Wallis rank-sum test with the post hoc test of the Tukey–Kramer method was applied. Values of *P* < 0.05 were considered statistically significant.

## Results

### Maternal chronic *Echinococcus multilocularis* infection alleviates colitis in C57BL/6 J mice

**Fig. 1:**
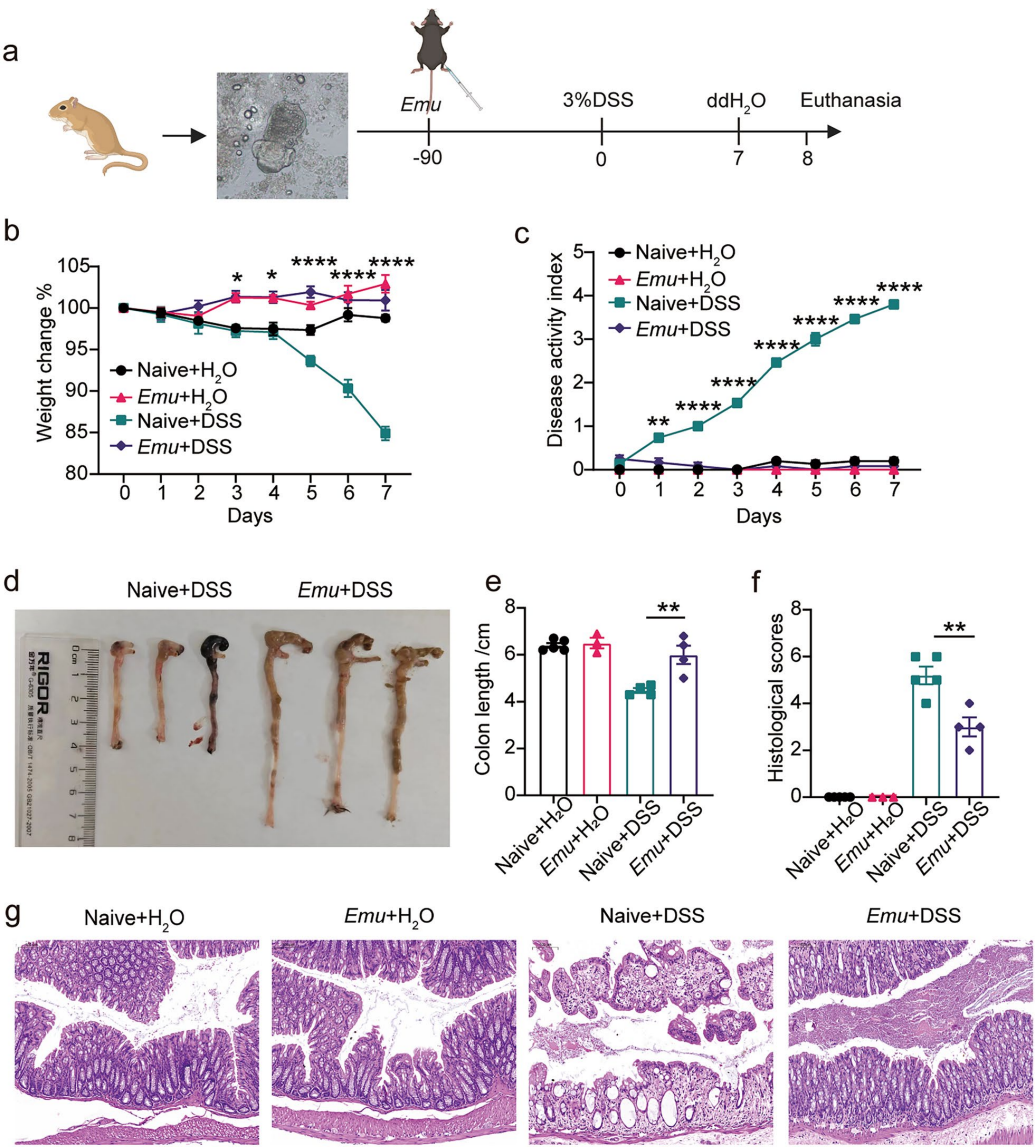
Maternal chronic *Echinococcus multilocularis* infection attenuates colitis in C57BL/6 J mice. **a**, The experiments were conducted on mice on day 90 postinfection (p.i.) of *Emu* in the peritoneal cavity and the colitis model establishment process. **b**, Change of body weight in maternal mice. **c**, Weight loss, stool consistency, and fecal blood were scored to provide the disease activity index (DAI) for each group. **d** and **e**, The colon length of each mouse. **f**, Inflammation scores estimated from distal colon tissue with hematoxylin and eosin (H&E) staining, and (**g**) representative H&E staining sections of distal colon tissues (×100; the scale bar represents 100 µm in length). Experiments were repeated twice with similar results. The data are shown from a representative experiment (mean ± SEM, standard error of the mean) . Statistical analysis was performed with two-way repeated analysis of variance (ANOVA) followed by Sidak’s posttest for panels **b** and **c**. Statistical analysis was performed with the Student’s *t*-test for panels **e** and **f** (*P < 0.05, **P < 0.01, ***P < 0.001, ****P < 0.0001)

Our recent publication demonstrated that Balb/c mice infected with *Emu* were protected against intestinal inflammation [18]. To assess the effect of *Emu* infection on maternal colitis, female mice were infected and subsequently treated with 3% DSS. The *Emu*-infected mice exhibited significantly less severe DSS-induced colitis compared with the naive mice, as evidenced by lower DAI scores (Fig. 1c), reduced weight loss (Fig. 1b), and preserved colon length (Fig. 1d and e). By contrast, DSS treatment resulted in uninfected female mice gaining a significantly higher DAI (characterized by diarrhea and blood in stool) score, less body weight, and shortened colon length. Furthermore, the pathological changes of DSS-induced colitis in maternal mice infected with *Emu* were investigated. Histological analysis revealed fewer signs of inflammation and crypt damage in the colon tissue of *Emu*-infected maternal mice (Fig. 1f and g). These findings demonstrate that maternal *Emu* infection confers resistance to DSS-induced colitis.

### Offspring of *Emu*-infected mice do not exhibit alleviated colitis compared with offspring of uninfected mice

**Fig. 2:**
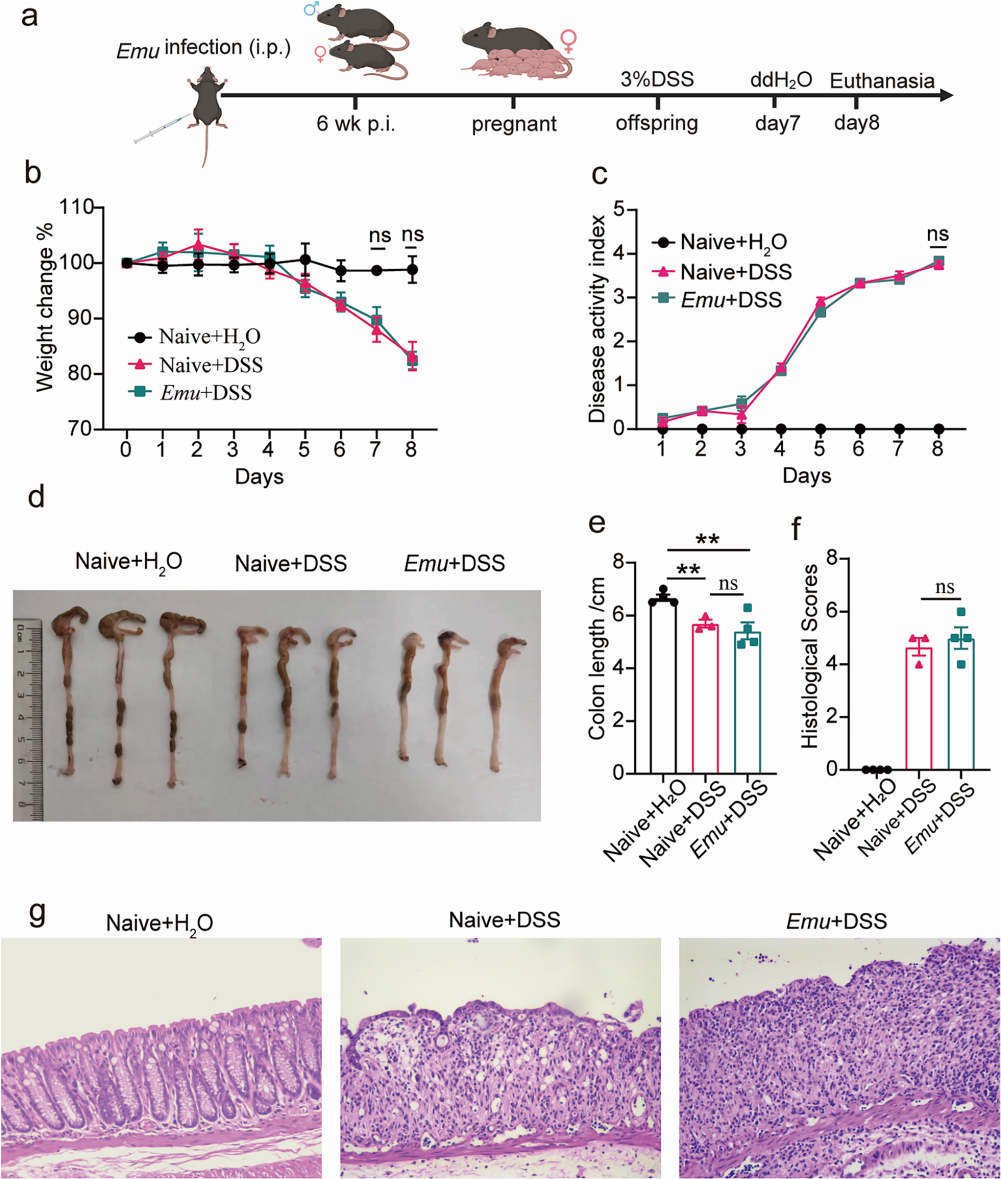
*Emu* infection-induced maternal phenotypic changes do not result in attenuated colitis in their offspring. **a**, Reproductive strategies of offspring mice and the establishment of the colitis model in offspring mice. **b**, Change of body weight in offspring mice. **c**, Disease activity index (DAI). **d** and **e**, colon length. **f**, The pathological scores and (**g**) representative H&E staining sections (× 100) of distal colon tissues. Data were shown for 3–4 mice per group. All results are expressed as mean ± SEM. Results are representative of two independent experiments. Statistical analysis was performed with two-way repeated ANOVA followed by Sidak’s posttest for panels **b** and **c**. Statistical analysis was performed with the Student’s *t*-test for panels **e** and **f** (*P < 0.05, **P < 0.01)

Helminth infections can affect maternal health and potentially have long-term impacts on the immune system, metabolism, and neurodevelopment of offspring. To investigate the transmission of maternal protection against colitis, offspring from *Emu*-infected and uninfected maternal mice were subjected to DSS-induced colitis. The offspring were weaned for 9 weeks, and a colitis model was established in offspring mice by administering 3% DSS via drinking water (Fig. 2a). Our results showed that the body weight of the naive + H_2_O group remained stable throughout the experiment. The offspring of *Emu*-infected maternal mice exhibited significant weight loss (Fig. 2b), higher DAI scores (Fig. 2c), and shorter colon lengths (Fig. 2d and e), similar to the uninfected mice offspring. Histological analysis also showed severe mucosal damage and inflammation (Fig. 2f and g). The naive + DSS and *Emu* + DSS groups showed damaged colon tissue, decreased numbers of goblet cells and crypts, altered crypt morphology, and significantly increased inflammatory cell infiltration. These results suggest that offspring mice from *Emu*-infected mice do not exhibit amelioration of DSS-induced colitis, indicating that the maternal protective effect against colitis is not inherited by the offspring.

### The upregulation of *Foxp3* in the postinfection maternal mouse colon is not transmitted to offspring

**Fig. 3:**
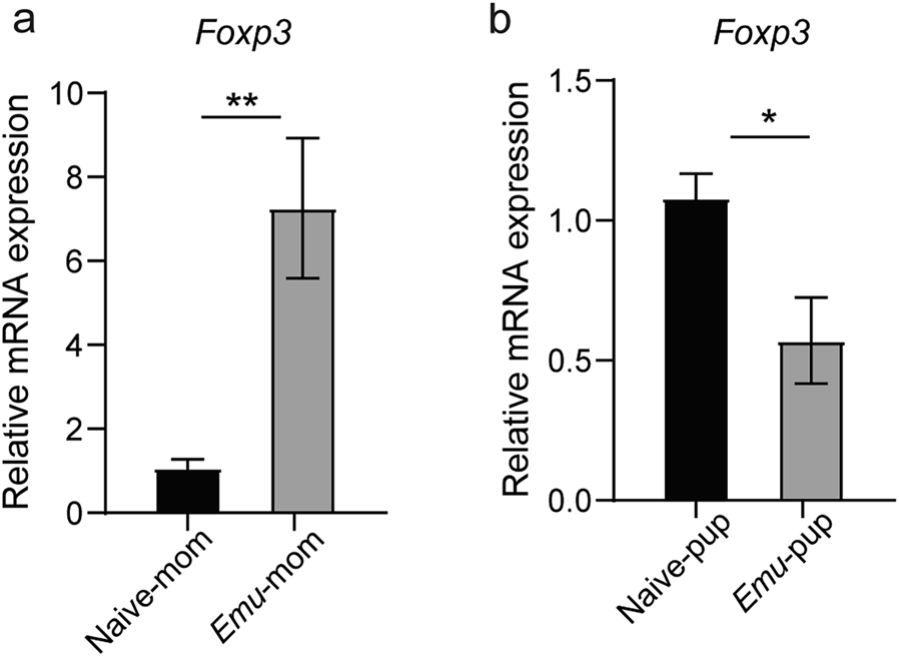
*Emu* infection promotes colonic *Foxp3* expression, but no increase is observed in the offspring. **a**, The relative expression of *Foxp3* in maternal mice. **b**, The relative expression of *Foxp3* in offspring mice. Data are shown for 3–5 samples per group. All results are expressed as the mean ± SEM. Results are representative of two independent experiments. Statistical analysis was performed with the Student’s *t*-test (*P < 0.05, **P < 0.01)

Tregs, characterized by the expression of Foxp3, play pivotal roles in helminth-mediated gut immune regulation by modulating immune responses, reducing inflammation, and maintaining gut homeostasis during helminth infections [18, 19]. We used qPCR to analyze the expression of *Foxp3* in colonic tissue in maternal mice 90 days post-*Emu* infection. Results showed that *Foxp3* expression was significantly higher in the *Emu*-mom group compared with the naive-mom group (*P* < 0.01, Fig. 3a). To further investigate the impact of *Emu* infection on the immune function of offspring mice, the *Foxp3* expression levels in colonic tissue were measured using qPCR. Results showed a decreasing trend in *Foxp3* expression in the offspring of the *Emu* mice (*P* < 0.05, Fig. 3b). This indicates that the maternal immune function induced by *Emu* infection was not transferred to the offspring.

### Maternal *Emu* infection alters gut microbiota composition in maternal mice and their uninfected offspring

**Fig. 4:**
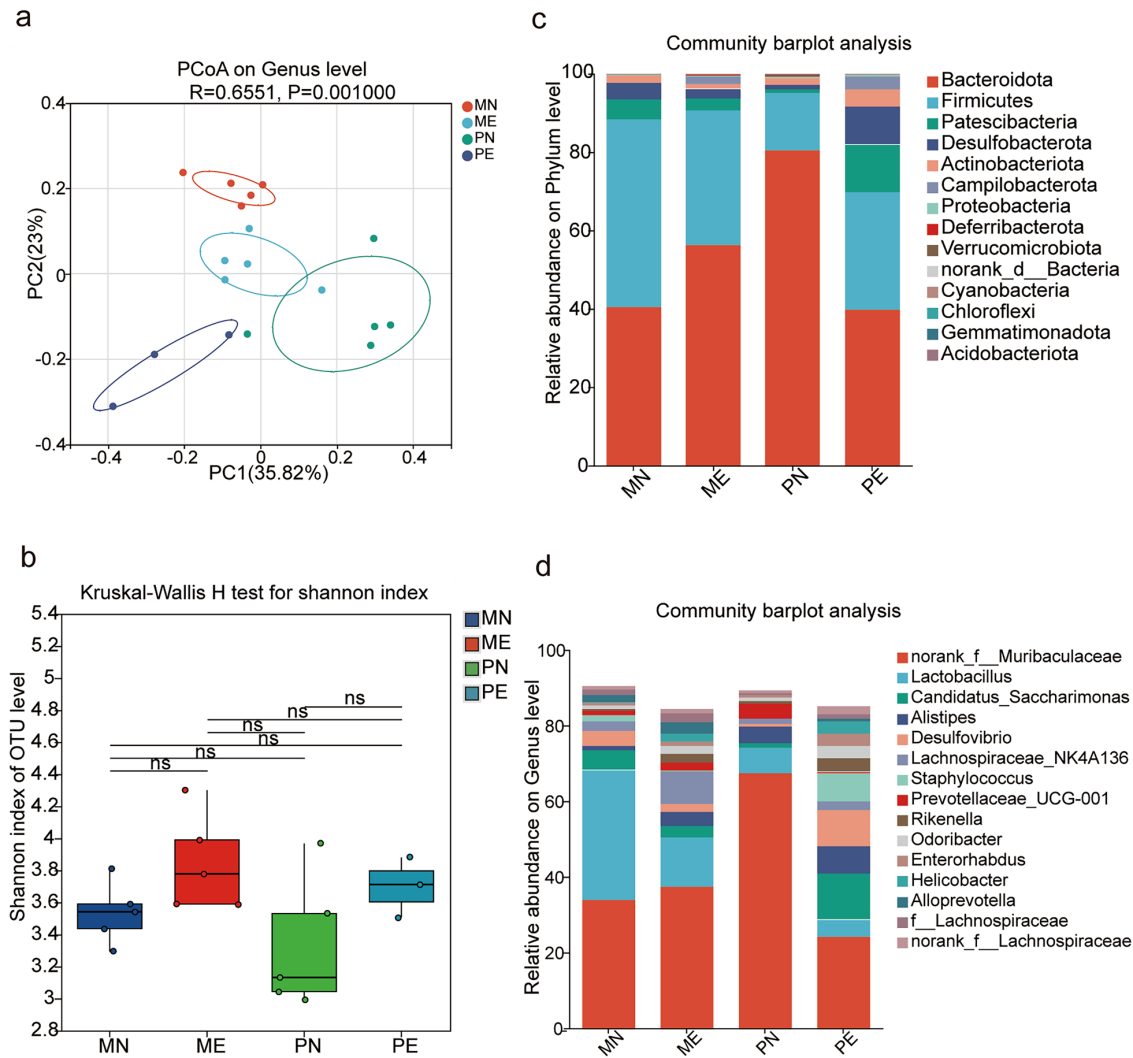
Maternal *Emu* infection induces alterations in gut microbiota homeostasis in both maternal mice and their uninfected offspring. **a**, Principal coordinate analysis (PCoA) for the gut microbiome of the samples based on Bray‒Curtis distance at the genus level (*R* = 0.6551, *P* = 0.001). **b**, Shannon index of alpha diversity (*P* > 0.05). **c**, The relative abundances of taxa at the phylum level. **d**, The relative abundances of taxa at the genus level. Data are shown for 3–5 mice per group. Statistical analysis was performed with the permutational multivariate analysis of variance (PERMANOVA) test for panel **a** and the two-sided Wilcoxon rank-sum test for panel **b**

The gut microbiota plays a key role in immune system development during early life, with maternal microbiota significantly influencing bacterial colonization in offspring. To determine whether maternal *Emu* infection alters the gut microbiota and affects the offspring, we analyzed the gut microbiota composition and microbial abundance in *Emu*-infected and naive maternal mice and their offspring using 16S rRNA sequencing. Bray–Curtis-based principal coordinate analysis (PCoA) revealed distinct clustering of microbiota composition between *Emu*-infected maternal mice and naive mice, In addition, the gut microbiota of offspring from *Emu*-infected mice exhibited significant changes compared with offspring of uninfected mice (Fig. 4a). However, alpha diversity, as measured by the Shannon index, did not show significant differences between groups (Fig. 4b). To further examine the structural variation of gut microbiota in *Emu*-infected mice and their offspring, differences were assessed across various taxonomic levels. At the phylum level, *Firmicutes* and *Bacteroidetes* were found to be the dominant flora in the intestinal tract of mice, the abundance of *Firmicutes* decreased and the abundance of *Bacteroidetes* increased in the *Emu*-infected mice (ME). However, in the offspring from *Emu*-infected mice (PE), the abundance of *Firmicutes* and *Bacteroidetes* showed opposing trends (Fig. 4c). At the genus level, the relative abundance of *Rikenella*, *Rikenellaceae_RC9_gut _group*, *Turicibacter*, and *Parabacteroides* was significantly higher in *Emu*-infected mice, whereas the abundance of *Lactobacillus*, *Staphylococcus*, and *Bifidobacterium* was lower compared with controls. In the offspring from *Emu*-infected mice, the relative abundances of *Candidatus Saccharimonas*, *Desulfovibrio*, *Odoribacter*, *Helicobacter*, *Erysipelotrichaceae*, and *Alloprevotella* were significantly increased, while *Muribaculum*, *Akkermansia*, and *Parabacteroides* were significantly decreased (Fig. 4d). These results suggest that maternal *Emu* infection leads to notable shifts in gut microbiota composition; these changes are reflected in the offspring, although the microbiota structure in maternal and offspring groups differs.

### Comparison of gut microbiota changes in maternal mice following *Emu* infection and their uninfected offspring

**Fig. 5:**
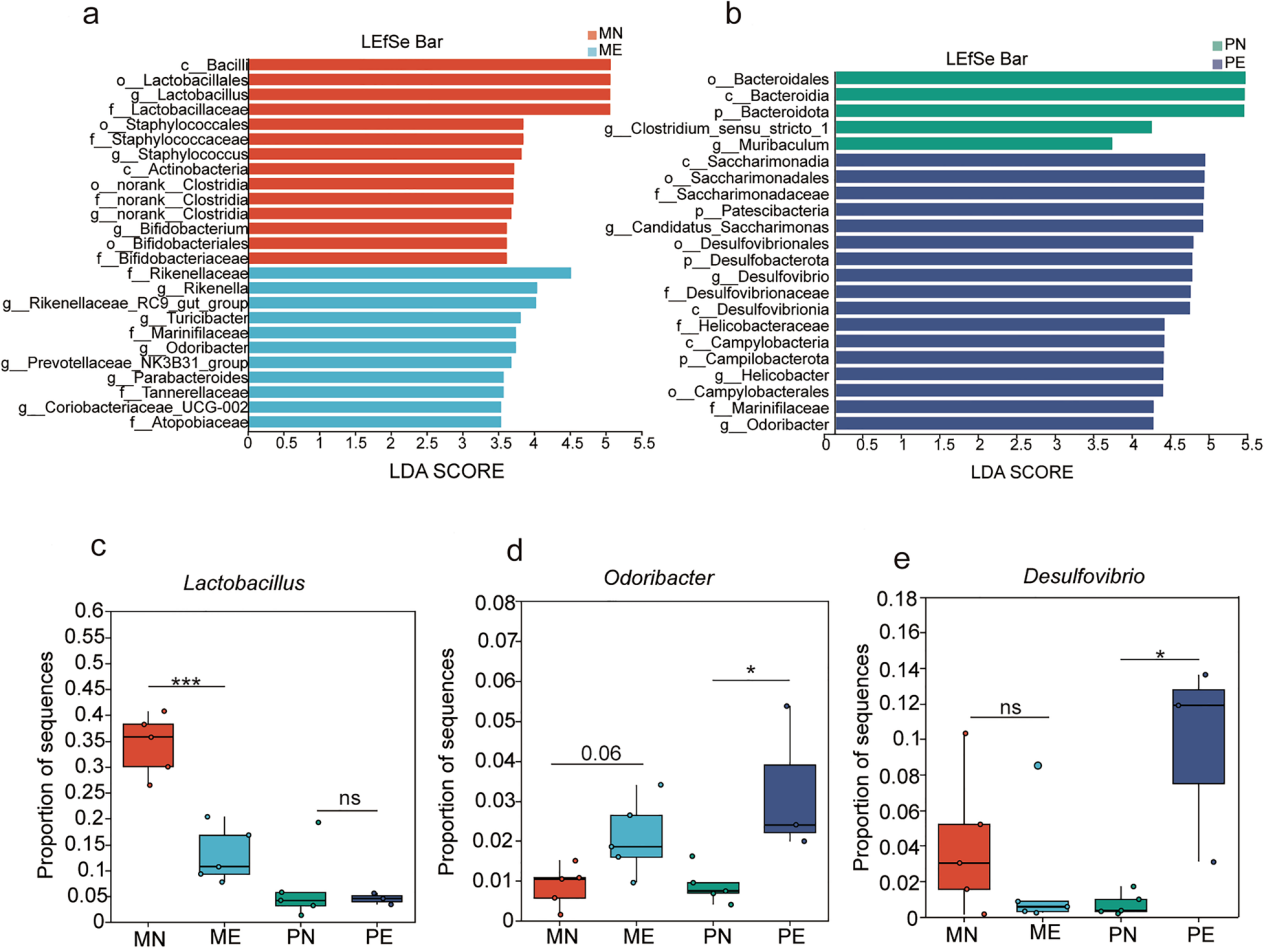
Linear discriminant analysis effect size (Lefse) analysis at the genus level in maternal mice and their uninfected offspring. **a**, LEfSe analysis comparing the gut microbiome of fecal samples from naive and *Emu*-infected maternal mice. **b**, LEfSe analysis comparing the gut microbiome of fecal samples from offspring of naive and Emu-infected mice. Only taxa with an linear discriminant analysis (LDA) score > 3.5 are shown. **c**, Statistical analysis of the differences in *Lactobacillus*. **d**, Statistical analysis of the differences in *Odoribacter*. **e**, Statistical analysis of the differences in *Desulfovibrio*. Statistical analyses were performed with the Wilcoxon rank-sum test for panels **c**, **d**, and **e**. (*P < 0.05, **P < 0.01, ***P< 0.001)

To identify differences between groups at the genus level, linear discriminant analysis effect size (LefSe) was used to determine differentially abundant genera with an linear discriminant analysis (LDA) score > 3.5. In Fig. 5a, *Emu* infection in maternal mice was linked to an increased relative abundance of the genera *Rikenella*, *Rikenellaceae_RC9_gut _group*, *Turicibacter*, *Odoribacter,*, and *Parabacteroides* within the gut microbiota and a decrease in *Lactobacillus*, *Staphylococcus*, and *Bifidobacterium* (Fig. 5a). In Fig. 5b, the gut microbiota of offspring from *Emu*-infected mice exhibited elevated levels of *Candidatus Saccharimonas*, *Desulfovibrio*, *Helicobacter*, and *Odoribacter*, while *Muribaculum* and *Clostridium_sensu_stricto_1* were significantly decreased (Fig. 5b). The maternal and offspring mice exhibited similar trends in the changes of certain bacterial genera. For example, *Odoribacter* was elevated in both *Emu*-infected maternal and offspring mice (Fig. 5d). At the same time, the anti-inflammatory genus *Lactobacillus* showed no significant differences in the offspring (Fig. 5c); the proinflammatory bacterium *Desulfovibrio* was significantly increased in the offspring of *Emu*-infected mice (Fig. 5e). These observations indicate that although the overall differential bacterial taxa between maternal and offspring mice were not identical, maternal helminth infection could partially influence the gut microbiota composition of the offspring.

## Discussion

The “hygiene hypothesis” proposes that reduced exposure to microbial infections in early life, especially in Western societies, contributes to the rise of allergies and autoimmune diseases [20]. In line with this hypothesis, emerging research indicates that helminth exposure may offer therapeutic benefits by promoting balanced immune responses. Although helminths elicit immune responses in their hosts, they also employ immune evasion mechanisms to avoid complete host immunity [21, 22]. Foxp3^+^ T regulatory cells play a crucial role in suppressing inflammation and maintaining tissue homeostasis [23]. Recent studies have shown that infection with parasitic helminths, such as *Echinococcus multilocularis* (*Emu*) [24], *Heligmosomoides polygyrus* [25], *Hymenolepis diminuta* [26], and so on, reduces chemically-induced colitis in mice, likely by modulating the host’s immune responses [27]. Consistent with these findings, our results revealed maternal *Emu* infection significantly alleviates colitis, as indicated by decreased weight loss, reduced DAI scores, and improved histopathological outcomes. Interestingly, emerging evidence suggests that *Foxp3* expression is not limited to T cells. For example, in certain disease contexts, *Foxp3* expression has also been identified in myeloid cells, including macrophages. Notably, Foxp3^+^ macrophages have been implicated in regulating poststroke neuroinflammation [28]. In our study, the observed elevated *Foxp3* expression further supports the notion that *Emu* infection induces a robust immune regulatory response, which contributes to the reduction of colonic inflammation. Overall, these results align with previous studies suggesting that helminth infections modulate immune responses to protect against inflammatory diseases, such as IBD.

Maternal perinatal programming is a critical period of developmental plasticity, where environmental factors such as infections can have lasting effects on offspring health [29]. Previous studies reported that offspring of helminth-infected dams developed enhanced immunity against gut infections and altered inflammatory responses [30]. However, when we examined the potential inheritance of the colitis-resistance phenotype in offspring, our results showed that the offspring of the *Emu*-infected maternal mice did not exhibit similar ameliorative effects when subjected to DSS-induced colitis. Both offspring of the infected and control group demonstrated significant weight loss, high DAI scores, and elevated histopathological scores without notable differences. In addition, the absence of significant differences in *Foxp3* expression levels in the offspring further supports the lack of heritability of the mother’s ameliorative phenotype. Various factors, including genetics, gut microbiota, diet, and environmental conditions could influence these findings.

The gut microbiota is crucial for host health, influencing immune function, metabolism, and behavior [31, 32]. Gut microbiota is largely shaped by early life environmental factors, prompting investigations into whether helminth infections affect not only the host but also their offspring [33]. The gut microbiota analysis provides insights into the potential mechanisms behind these observations. Our study revealed that maternal infection with *Emu* induced significant alterations in gut microbiota of maternal mice compared with naive mice. Specifically, the abundance of several bacterial genera, such as *Rikenella*, *Rikenellaceae_RC9_gut_group*, *Turicibacter*, *Odoribacter*, and *Parabacteroides*, was increased in the *Emu*-infected mice. Conversely, the abundance of genera such as *Lactobacillus*, *Staphylococcus*, and *Bifidobacterium* was reduced following *Emu* infection. These results highlight the profound impact of maternal *Emu* infection on gut microbiota composition, which aligns with previous studies showing that helminths can modulate gut microbial communities, influencing immune responses and potentially shaping long-term health outcomes [18, 34]. Interestingly, while the maternal microbiota exhibited significant changes, gut microbiota structure of the offspring from *Emu*-infected mice displayed a distinctly different pattern. In the offspring from *Emu*-infected mice, *Candidatus Saccharimonas*, *Desulfovibrio*, *Helicobacter*, and *Odoribacter* were significantly more abundant, whereas genera such as *Muribaculum* and *Clostridium_sensu_stricto_1* were significantly depleted. This suggests that, despite the maternal microbiota changes, the microbiota of offspring was markedly different and did not fully mirror the maternal microbial profile. These findings suggest that maternal infection-induced shifts in microbiota may not be fully transmitted to offspring, potentially owing to developmental or environmental factors influencing microbiota establishment in the offspring’s gut. Despite significant differences in the gut microbiota structures of maternal and offspring mice, several similar trends were noted in both infected group and offspring mice, such as the increase in *Odoribacter* and *Rikenella*. These trends indicated that maternal gut microbiota alterations could partially influence the offspring’s microbiota composition.

Our findings reveal both consistencies and discrepancies when compared with previously published studies on helminth-induced microbiota alterations. Specifically, our results align with prior studies showing increased Bacteroidota abundance in *H. polygyrus*-infected mice [10, 35]. However, the observed reduction in *Lactobacillus* in C57BL/6 mice contrasts with a study indicating that *Emu* infection increased the abundance of *Lactobacillus* in Balb/c mice [18]. The role of *Lactobacillus* in regulating immune responses is well-documented, particularly for its anti-inflammatory effects [36]. Our unpublished data from the lab indicate that such differences may be species-dependent and influenced by baseline gut microbiomes and the specific stages of helminth infection. This highlights the complexity of microbiota inheritance and its dependence on various factors beyond maternal infection alone, including the origin of the mice used in the study. This is supported by our subsequent unpublished data, which show that even when using mice with the same genetic background but obtained from different facilities, infection with *Emu* results in distinct gut microbial responses, especially in terms of *Lactobacillus* abundance. These findings strongly suggest that variations in baseline microbiota significantly influence the direction of microbial shifts following helminth infection. In addition, as shown in Fig. 4, we also observed differences in the baseline gut microbiota compositions between uninfected maternal and their offspring mice, further supporting the notion. These observations may help explain the discrepancies in the microbial shifts between maternal and offspring mice. *E. multilocularis* as a tissue-dwelling helminth has been extensively studied for its capacity to modulate host immune responses [37]. Recent studies have shown that *E. multilocularis* infection significantly alleviates DSS-induced colitis in mice by suppressing Th1/Th17-mediated inflammatory responses [24]. In addition, research has found that the helminth-derived protein can mimic the function of human transforming growth factor (TGF)-β, inducing the activation of regulatory T cells [7]. Our previous findings also indicated that a serine protease inhibitor secreted by *E. multilocularis* (*Emu*-serpin) has been shown to mitigate colonic inflammation by reducing tissue damage and lowering the levels of proinflammatory cytokines in the colon [16]. It is possible that the observed effects are due to the excretory-secretory products released by the *Emu* during infection. This mechanism could potentially explain the observed amelioration of colitis in maternal mice following *Emu* infection. In addition, changes in specific microbial taxa may be involved. *Odoribacter* belongs to the Bacteroidota phylum and is generally considered a short-chain fatty acid (SCFA)-producing genus. Several studies have reported that the abundance of *Odoribacter*, particularly *Odoribacter splanchnicus*, is often negatively correlated with colonic inflammation, implying a potential protective role in maintaining gut homeostasis [38, 39]. Although *Odoribacter* increased in maternal and offspring mice, only the maternal mice exhibited reduced colitis susceptibility. This suggests that the presence of *Odoribacter* alone may not be sufficient to confer protection. Conversely, the notable increase in *Desulfovibrio* in offspring, a genus linked to IBD pathology [40, 41], may exacerbate inflammation, counteracting any inherited benefits from maternal microbiota. These findings underscore the complexity of host–microbiota interactions.

Nevertheless, this study has several limitations that should be considered. The establishment of *E. multilocularis* infection in the peritoneal cavity was associated with reduced reproductive performance in infected dams, leading to a limited number of offspring available for subsequent analyses. This biological constraint may have affected the sample size and reduced the statistical power of offspring-related observations. In addition, although the results indicate a potential association among *Foxp3* upregulation, microbiota alterations, and reduced colitis severity in maternal mice, the precise molecular and cellular mechanisms mediating these effects remain incompletely understood. We need to include more makers and clarify the potential mechanism involved.

## Conclusions

The results from this study demonstrated that maternal mice exhibited significant resistance to colitis, which was associated with an increased expression of *Foxp3* following *Emu* infection. However, this protective phenotype was not transmitted to the offspring via gut microbiota inheritance. Comparative analysis of gut microbiota between maternal and offspring mice revealed substantial structural differences, indicating a relatively weak maternal influence on the offspring’s gut microbial composition. The failure of the offspring to inherit immune-modulating bacteria from their mothers likely contributed to their inability to mitigate colitis through microbiota-mediated immune regulation. Consequently, maternal resistance to colitis depends on other factors, potentially including direct stimulation by *Emu* or other nonmicrobial mechanisms. Further investigation is required to elucidate these underlying processes. These findings provide a foundation for developing targeted therapies that leverage helminth-induced immune modulation, although intergenerational immune regulation remains a complex challenge.

## Data Availability

The raw sequencing data in this study have been deposited at NCBI under BioProject numbers PRJNA1291470. Other relevant data supporting the findings of this study are available from the corresponding authors upon reasonable request.

## Abbreviations

*Emu*: *Echinococcus multilocularis*
DSS: Dextran sulfate sodium
IBD: Inflammatory bowel disease
Foxp3: Forkhead box protein P3
SPF: Specific-pathogen-free
PSCs: Protoscoleces
PBS: Phosphate-buffered saline
DAI: Disease activity index
H&E: Hematoxylin and eosin
LP: Lamina propria
qPCR: Real-time fluorescence quantitative polymerase chain reaction
PCoA: Principal coordinate analysis
LefSe: Linear discriminant analysis effect size
LDA: Linear discriminant analysis
OTU: Operational taxonomic units
ME: The *Emu*-infected mice
MN: The uninfected mice
PE: The offspring from the *Emu*-infected mice
PN: The offspring from the uninfected mice

## References

[1] Hotez PJ, Brindley PJ, Bethony JM, King CH, Pearce EJ, Jacobson J. Helminth infections: the great neglected tropical diseases. J Clin Invest. 2008;118:1311–21. 18382743 10.1172/JCI34261 PMC2276811

[2] Erb KJ. Can helminths or helminth-derived products be used in humans to prevent or treat allergic diseases? Trends Immunol. 2009;30:75–82. 19138565 10.1016/j.it.2008.11.005

[3] Logan J, Navarro S, Loukas A, Giacomin P. Helminth-induced regulatory T cells and suppression of allergic responses. Curr Opin Immunol. 2018;54:1–6. 29852470 10.1016/j.coi.2018.05.007

[4] Elliott DE, Urban JFJR, Argo CK, Weinstock JV. Does the failure to acquire helminthic parasites predispose to Crohn’s disease? FASEB J. 2000;14:1848–55. 10973934 10.1096/fj.99-0885hyp

[5] Maizels RM, McSorley HJ. Regulation of the host immune system by helminth parasites. J Allergy Clin Immunol. 2016;138:666–75. 27476889 10.1016/j.jaci.2016.07.007 PMC5010150

[6] Gazzinelli-Guimaraes PH, Nutman TB. Helminth parasites and immune regulation. F1000Res. 2018;7:1685. 10.12688/f1000research.15596.1 PMC6206608 30416709

[7] White MPJ, McManus CM, Maizels RM. Regulatory T-cells in helminth infection: induction, function and therapeutic potential. Immunology. 2020;160:248–60. 32153025 10.1111/imm.13190 PMC7341546

[8] Dominguez-Villar M, Hafler DA. Regulatory T cells in autoimmune disease. Nat Immunol. 2018;19:665–73. 29925983 10.1038/s41590-018-0120-4 PMC7882196

[9] Lu L, Barbi J, Pan F. The regulation of immune tolerance by FOXP3. Nat Rev Immunol. 2017;17:703–17. 28757603 10.1038/nri.2017.75 PMC5793224

[10] Su C, Su L, Li Y, Long SR, Chang J, Zhang W, et al. Helminth-induced alterations of the gut microbiota exacerbate bacterial colitis. Mucosal Immunol. 2018;11:144–57. 28352104 10.1038/mi.2017.20 PMC5620113

[11] Gaillard R, Santos S, Duijts L, Felix JF. Childhood health consequences of maternal obesity during pregnancy: a narrative review. Ann Nutr Metab. 2016;69:171–80. 27855382 10.1159/000453077

[12] Su C-W, Chen C-Y, Mao T, Chen N, Steudel N, Jiao L, et al. Maternal helminth infection protects offspring from high-fat-diet-induced obesity through altered microbiota and SCFAs. Cell Mol Immunol. 2023;20:389–403. 36788341 10.1038/s41423-023-00979-1 PMC10066288

[13] Torres J, Hu J, Seki A, Eisele C, Nair N, Huang R, et al. Infants born to mothers with IBD present with altered gut microbiome that transfers abnormalities of the adaptive immune system to germ-free mice. Gut. 2020;69:42–51. 31036757 10.1136/gutjnl-2018-317855

[14] Liu Z, Guo X, Guo A, Zhang S, Zou Y, Wang Y, et al.. HIV protease inhibitor nelfinavir is a potent drug candidate against echinococcosis by targeting Ddi1-like protein. EBioMedicine. 2022;82:104177. 35843171 10.1016/j.ebiom.2022.104177 PMC9294487

[15] Spiliotis M, Brehm K. Axenic in vitro cultivation of Echinococcus multilocularis metacestode vesicles and the generation of primary cell cultures. Methods Mol Biol. 2009;470:245–62. 19089387 10.1007/978-1-59745-204-5_17

[16] Li X, Liu Y, Zou Y, Zhang J, Wang Y, Ding Y, et al. *Echinococcus multilocularis* serpin regulates macrophage polarization and reduces gut dysbiosis in colitis. Infect Immun. 2024;92:e0023224. 39037247 10.1128/iai.00232-24 PMC11320943

[17] Smith P, Mangan NE, Walsh CM, Fallon RE, McKenzie ANJ, van Rooijen N, et al. Infection with a helminth parasite prevents experimental colitis via a macrophage-mediated mechanism. J Immunol. 2007;178:4557–66. 17372014 10.4049/jimmunol.178.7.4557

[18] Wang Y, Guo A, Zou Y, Mu W, Zhang S, Shi Z, et al. Interaction between tissue-dwelling helminth and the gut microbiota drives mucosal immunoregulation. NPJ Biofilms Microbiomes. 2023;9:43. 37355675 10.1038/s41522-023-00410-7 PMC10290639

[19] Rapin A, Harris NL. Helminth-bacterial interactions: cause and consequence. Trends Immunol. 2018;39:724–33. 29941203 10.1016/j.it.2018.06.002

[20] Yazdanbakhsh M, Matricardi PM. Parasites and the hygiene hypothesis: regulating the immune system? Clin Rev Allergy Immunol. 2004;26:15–24. 14755072 10.1385/CRIAI:26:1:15

[21] Maizels RM, Pearce EJ, Artis D, Yazdanbakhsh M, Wynn TA. Regulation of pathogenesis and immunity in helminth infections. J Exp Med. 2009;206:2059–66. 19770272 10.1084/jem.20091903 PMC2757871

[22] Sorobetea D, Svensson-Frej M, Grencis R. Immunity to gastrointestinal nematode infections. Mucosal Immunol. 2018;11:304–15. 29297502 10.1038/mi.2017.113

[23] Laukova M, Glatman ZA. Regulatory T cells as a therapeutic approach for inflammatory bowel disease. Eur J Immunol. 2023;53:e2250007. 36562391 10.1002/eji.202250007 PMC10107179

[24] Wang J, Goepfert C, Mueller N, Piersigilli A, Lin R, Wen H, et al. Larval Echinococcus multilocularis infection reduces dextran sulphate sodium-induced colitis in mice by attenuating T helper type 1/type 17-mediated immune reactions. Immunology. 2018;154:76–88. 29121394 10.1111/imm.12860 PMC5904711

[25] Hang L, Blum AM, Setiawan T, Urban JP Jr, Stoyanoff KM, Weinstock JV. Heligmosomoides polygyrus bakeri infection activates colonic Foxp3^+^ T cells enhancing their capacity to prevent colitis. J Immunol. 2013;191:1927–34. 23851695 10.4049/jimmunol.1201457 PMC3790665

[26] Wang A, Arai T, Campbell A, Reyes JL, Lopes F, McKay DM. Triggering immunological memory against the tapeworm Hymenolepis diminuta to protect against colitis. Parasite Immunol. 2017;39:12490. 10.1111/pim.12490 28892562

[27] Chu KM, Watermeyer G, Shelly L, Janssen J, May TD, Brink K, Benefeld G, et al. Childhood helminth exposure is protective against inflammatory bowel disease: a case control study in South Africa. Inflamm Bowel Dis. 2013;19:614–20. 23380935 10.1097/MIB.0b013e31827f27f4

[28] Cai W, Hu M, Li C, Wu R, Lu D, Xie C, et al. FOXP3+ macrophage represses acute ischemic stroke-induced neural inflammation. Autophagy. 2023;19:1144–63. 36170234 10.1080/15548627.2022.2116833 PMC10012925

[29] Villanueva-Ortega E, Garcés-Hernández MJ, Garibay Nieto GN. Pre- and post-natal nutritional factors in the metabolic regulation of obesity. Rev médica Hosp Gen Méx. 2017;80:111–8.

[30] Lim AI, McFadden T, Link VM, Han S-J, Karlsson R-M, Stacy A, et al. Prenatal maternal infection promotes tissue-specific immunity and inflammation in offspring. Science. 2021;373:3002. 10.1126/science.abf3002 34446580

[31] O’Hara AM, Shanahan F. The gut flora as a forgotten organ. EMBO Rep. 2006;7:688–93. 16819463 10.1038/sj.embor.7400731 PMC1500832

[32] de Vos WM, Tilg H, Van Hul M, Cani PD. Gut microbiome and health: mechanistic insights. Gut. 2022;71:1020–32. 35105664 10.1136/gutjnl-2021-326789 PMC8995832

[33] Sommer F, Bäckhed F. The gut microbiota–masters of host development and physiology. Nat Rev Microbiol. 2013;11:227–38. 23435359 10.1038/nrmicro2974

[34] Fricke WF, Song Y, Wang A-J, Smith A, Grinchuk V, Mongodin E, et al. Type 2 immunity-dependent reduction of segmented filamentous bacteria in mice infected with the helminthic parasite Nippostrongylus brasiliensis. Microbiome. 2015;3:40. 26377648 10.1186/s40168-015-0103-8 PMC4574229

[35] Rausch S, Held J, Fischer A, Heimesaat MM, Kühl AA, Bereswill S, et al. Small intestinal nematode infection of mice is associated with increased enterobacterial loads alongside the intestinal tract. PLoS ONE. 2013;8:e74026. 24040152 10.1371/journal.pone.0074026 PMC3769368

[36] Liu H-Y, Gu F, Zhu C, Yuan L, Zhu C, Zhu M, et al. Epithelial heat shock proteins mediate the protective effects of in dextran sulfate sodium-induced colitis. Front Immunol. 2022;13:865982. 35320932 10.3389/fimmu.2022.865982 PMC8934773

[37] Zou Y, Pu L, Guo A, Li Y, Liu Y, Wang Y, et al. Helminth reshapes host gut microbiota and immunoregulation by deploying an antimicrobial program of innate immunity. Gut Microbes. 2025;17:2496447. 40266093 10.1080/19490976.2025.2496447 PMC12026035

[38] Morgan XC, Tickle TL, Sokol H, Gevers D, Devaney KL, Ward DV, Reyes JA, et al. Dysfunction of the intestinal microbiome in inflammatory bowel disease and treatment. Genome Biol. 2012;13:R79. 23013615 10.1186/gb-2012-13-9-r79 PMC3506950

[39] Hiippala K, Barreto G, Burrello C, Diaz-Basabe A, Suutarinen M, Kainulainen V, et al. Novel strain and its outer membrane vesicles exert immunoregulatory effects. Front Microbiol. 2020;11:575455. 33281770 10.3389/fmicb.2020.575455 PMC7689251

[40] Fite A, Macfarlane S, Furrie E, Bahrami B, Cummings JH, Steinke DT, et al. Longitudinal analyses of gut mucosal microbiotas in ulcerative colitis in relation to patient age and disease severity and duration. J Clin Microbiol. 2013;51:849–56. 23269735 10.1128/JCM.02574-12 PMC3592070

[41] Humbel F, Rieder JH, Franc Y, Juillerat P, Scharl M, Misselwitz B, et al. Association of alterations in intestinal microbiota with impaired psychological function in patients with inflammatory bowel diseases in remission. Clin Gastroenterol Hepatol. 2020;18:2019-29.e11. 31546058 10.1016/j.cgh.2019.09.022

